# Genome-wide association mapping in elite winter wheat breeding for yield improvement

**DOI:** 10.1007/s13353-023-00758-8

**Published:** 2023-04-29

**Authors:** Mirosław Tyrka, Paweł Krajewski, Piotr Tomasz Bednarek, Kinga Rączka, Tadeusz Drzazga, Przemysław Matysik, Róża Martofel, Urszula Woźna-Pawlak, Dorota Jasińska, Małgorzata Niewińska, Bogusława Ługowska, Dominika Ratajczak, Teresa Sikora, Edward Witkowski, Ada Dorczyk, Dorota Tyrka

**Affiliations:** 1 Department of Biotechnology and Bioinformatics, Rzeszow University of Technology, Powstańców Warszawy 6, 35-959 Rzeszów, Poland; 2 Institute of Plant Genetics, Polish Academy of Sciences, Strzeszyńska 34, 60-479 Poznań, Poland; 3 Plant Breeding and Acclimatization Institute – National Research Institute, Radzików, 05-870 Błonie, Poland; 4 Małopolska Plant Breeding Ltd, Sportowa 21, 55-040 Kobierzyce, Poland; 5 Plant Breeding Strzelce Group IHAR Ltd, Główna 20, 99-307 Strzelce, Poland; 6 Poznań Plant Breeding Ltd, Kasztanowa 5, 63-004 Tulce, Poland; 7 DANKO Plant Breeders Ltd, Ks. Strzybnego 23, 47-411 Rudnik, Poland; 8 Plant Breeding Smolice Ltd, Smolice 146, 63-740 Kobylin, Poland

**Keywords:** Marker-trait associations, Genome-wide association studies, Single-nucleotide polymorphisms, Yield

## Abstract

Increased grain yield (GY) is the primary breeding target of wheat breeders. We performed the genome-wide association study (GWAS) on 168 elite winter wheat lines from an ongoing breeding program to identify the main determinants of grain yield. Sequencing of Diversity Array Technology fragments (DArTseq) resulted in 19,350 single-nucleotide polymorphism (SNP) and presence-absence variation (PAV) markers. We identified 15 main genomic regions located in ten wheat chromosomes (1B, 2B, 2D, 3A, 3D, 5A, 5B, 6A, 6B, and 7B) that explained from 7.9 to 20.3% of the variation in grain yield and 13.3% of the yield stability. Loci identified in the reduced genepool are important for wheat improvement using marker-assisted selection. We found marker-trait associations between three genes involved in starch biosynthesis and grain yield. Two starch synthase genes (*TraesCS2B03G1238800* and *TraesCS2D03G1048800*) and a sucrose synthase gene (*TraesCS3D03G0024300*) were found in regions of *QGy.rut-2B.2*, *QGy.rut-2D.1*, and *QGy.rut-3D*, respectively. These loci and other significantly associated SNP markers found in this study can be used for pyramiding favorable alleles in high-yielding varieties or to improve the accuracy of prediction in genomic selection.

**Supplementary Information:**

The online version contains supplementary material available at 10.1007/s13353-023-00758-8.

## Introduction  

Cultivating common wheat (*Triticum aestivum* L.) provides about 20% of the total calories used by the human population (Rasheed et al. 2018). Worldwide wheat harvest area exceeds 213 million ha, and about 28% of this area is located in Europe, including over 2.3 million ha in Poland (FAOSTAT 2022). However, there are limitations to the territorial expansion of wheat cultivation, and to meet the challenge of doubling the wheat yield by 2050 (Rasheed et al. 2018), significant yield increase per unit of area is required. To meet this challenge, increased genetic diversity deposited in landraces (Vikram et al. 2016), synthetic wheat varieties (Li et al. 2018), and wild relatives (Rasheed et al. 2018) needs to be identified and exploited in modern wheat cultivars, besides agronomical practices for yield improvement. The sequencing of the 17 Gb allohexaploid wheat (AABBDD) genome of Chinese Spring paved the way for genome-wide association studies (GWAS) and genomic selection in common wheat (Lukaszewski et al. 2014; Appels et al. 2018).

The wheat reference sequence provided a physical framework for mapping previously developed genetic markers with known sequences (Alaux et al. 2018), and marker sequences deposited in databases can be used to find regions with target genes (Tyrka et al. 2021b). Hybridization arrays or next-generation sequencing (NGS) are the most common ways to find single-nucleotide polymorphisms (SNPs) and presence–absence variations (PAVs). With the continuous development of new high-throughput NGS methods, the application of genotyping by sequencing (GBS) technologies (e.g., DArTseq) is considered to be the cost-efficient genotyping alternative (Jia et al. 2018) for genomics-based breeding (Poland et al. 2012). GBS gives the genetic information needed to determine economically significant marker-trait associations and develop new wheat cultivars.

Two main approaches to dissecting the genetic basis of complex quantitative traits in crop plants are genome-wide association studies (GWAS) and quantitative trait loci (QTL) mapping. Many QTLs associated with yield-related traits in bread wheat have been identified in biparental populations (Jin et al. 2020; Isham et al. 2021; Kang et al. 2021; Li et al. 2022). At present, GWAS has become more frequently used as it allows for the identification of parts of the complex, essential traits valid in a studied panel of genotypes (Neumann et al. 2011; Sukumaran et al. 2018; Liu et al. 2018; Garcia et al. 2019; Qaseem et al. 2019; Sheoran et al. 2019; Akram et al. 2021). Regions associated with grain yield and its component traits in wheat have been identified in drought and irrigated production conditions (Golabadi et al. 2011; Neumann et al. 2011; Assanga et al. 2017; Bhusal et al. 2017; Li et al. 2019; Khan et al. 2022). Haplotypes found in GWAS (Sehgal et al. 2020) linked to GY are also needed to map candidate genes (Nadolska-Orczyk et al. 2017).

Increased GY is the primary breeding purpose of wheat. The environment strongly influences GY and can be dissected into numerous traits related to phenology and kernel development. Major QTLs significantly associated with yield are currently the target for cloning, and comparative analysis of yield-related traits revealed 145 meta-QTLs and candidate genes (Yang et al. 2021). One of the leading environmental factors influencing yield is nitrogen availability. Nitrogen fertilizer, often used to increase production per unit area, can cause lodging. In wheat, lodging generally occurs after the flowering stage and can affect both the grain yield and the quality of the wheat. Lodging can also be caused by environmental factors, diseases, or pests affecting stems or roots (Keller et al. 1999). In wheat, stem characteristics such as material strength based on lignin concentration and stem thickness play a role (Berry et al. 2007; Berry and Berry 2015; Dreccer et al. 2020, 2022; Piñera-Chavez et al. 2016).

Depending on the population studied, GWAS can identify different genome regions responsible for shaping a trait (Yang et al. 2021). By introducing varieties with very different yield potential into the analyzed population, regions with major effects can be identified. Under long-term selection, polymorphism in these regions may have been lost, and other regions may contribute to yield. GWAS analysis of advanced breeding lines provides an opportunity to identify loci responsible for yield in a narrow gene pool and should indicate the main selection goals that can be achieved using marker-assisted selection. The present study aimed to identify the genomic region(s) associated with grain yield (GY) and component traits, i.e., coefficients of yield stability (STA), days to heading (DTH), plant height (PH), lodging (LDG), and thousand kernel weight (TKW) in a panel of elite wheat genotypes in a range of environments through the GWAS approach.

## Material and methods

### Phenotypic data collection and analysis

**Fig. 1:**
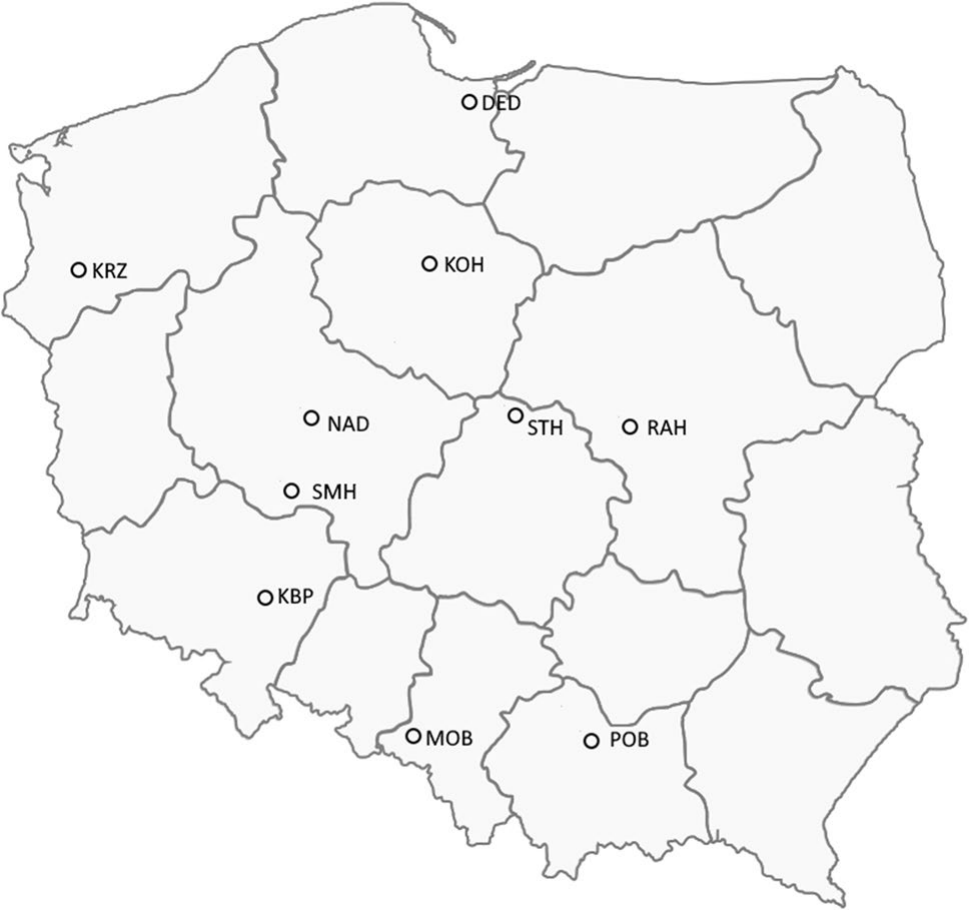
Distribution of experimental stations in Poland. DED—Dębina, KBP—Kobierzyce, KOH—Kończewice, KRZ—Krzemlin, MOB—Modzurów, NAD—Nagradowice, POB—Polanowice, RAH—Radzików, SMH—Smolice, and STH—Strzelce

Plant material included 168 breeding lines of common winter wheat and three cultivars evaluated in pre-registration trials in the 2019/2020 season (Table S1). The lines were planted at ten research stations located at Dębina (DED, N54°7′40″, E19°2′7″), Kobierzyce (KBP, N50°58′34″, E16°55′53″), Kończewice (KOH, N53°11′5″, E18°33′15″), Krzemlin (KRZ, N53°4′30″, E14°52′48″), Modzurów (MOB, N50°9′21″, E18°7′38″), Nagradowice (NAD, N52°19′4″, E17°9′1.7″), Polanowice (POB, N50°12′25″, E20°5′5″), Radzików (RAH, N52°12′53″, E20°38′45″), Smolice (SMH, N51°41′58″, E17°10′29″), and Strzelce (STH, N52°18′52″, E19°24′20″) dispersed across Poland (Fig. 1). Weather data indicate low rainfalls in March and April in most of the sites (Figure S1). The experiments were set up in a split-block design in three sets of 56 with three reference cultivars (Artist, Patras, and RGT Kilimanjaro) and 21 incomplete blocks per set. Each block consisted of 8 or 9 randomly assigned genotypes, accounting for three repetitions per genotype. The yield was measured for a 10-m^2^ plot (8 rows, 12.5 cm apart, and 10 m long). Only the inner six rows were harvested to avoid edge effects. Two agrotechnical levels were used. At the standard level (A1), the way the plants were grown and fertilized was the same as what was done for production at the respective experimental stations. At the intensive level (A2), nitrogen fertilization was increased by 40 kg/ha compared to level A1, and the plants were protected from disease and lodging. Experiments on the A1 level were conducted at five stations: DED, NAD, POB, RAH, and STH. Yield at the A2 level was measured at KBP, KOH, KRZ, MOB, and SMH stations. Grain yield was recorded along with four traits (Table 1). Grain yield was compared with the average values of three high-yielding reference cultivars (Artist, Patras, and RGT Kilimanjaro) referred to as a base of 100% (GY%). For yield, coefficients of stability (STA) were also calculated and used on GWAS to determine loci responsible for reducing environment-specific effects (see the “Data analysis” section below).

**Table 1:** Agronomical and morpho-physiological traits analyzed in ten sites in 2020 year

Trait	Abbreviation	Method of measurements	Unit/scale
Grain yield	GY	Measured after harvesting at 15% moisture	t·ha^−1^
Days to heading	DTH	Number of days from emergence (1st Jan) to awn appearance in 50% of the plants in a plot	Days
Lodging	LDG	Measured using a visual score from not logged (0) to completely logged (9)	Scale 1–9
Plant height	PH	Measured from ground level to the base of the spike at physiological maturity	cm
Thousand kernel weight	TKW	Measured as the average of three samples of 100 kernels	g

### Genotyping and annotations

DArTseq technology (Diversity Arrays Technology Pty Ltd., Bruce, Australia) was used for genotyping 171 winter wheat lines. Markers with minor allele frequencies below 0.05 and over 25% of missing data were removed. The genotyping resulted in 11,117 dominant type silicoDArTs (identified by the presence or absence of the whole target marker sequence) and 8233 SNPs. Most of the genomic and marker data for wheat was annotated on Chinese Spring IWGSC v1.0, and DArT sequences were mapped to the updated reference IWGSC v2.1 at URGI. Based on the BLAST e-score values for the 1.0E-05 threshold, markers’ locations were labeled as unique, most likely, homologous, or missing. BLAST of selected DArTseq markers vs. winter (Julius, Jagger, Arina, Mattis, Mace, Norin61, Robigus, and Clair) and spring (Weebill, Lancer, Stanley, Paragon, Spelt, Cadenza, Landmark) wheat from the pangenome project (Walkowiak et al. 2020) was performed on the Galaxy platform (Afgan et al. 2018) accessible at IPK Gatersleben (https://galaxy-web.ipk-gatersleben.de/). Additionally, markers were mapped to recently sequenced wheat cultivars (Renan_2.1, Zhang1817, Attraktion, Kariega, Fielder) at NCBI (The National Center for Biotechnology Information).

### Data analysis

Data were first processed using the Statistica 13.3 software (Tibco, CA, USA). The distribution of the data was checked with the Shapiro–Wilk test. The data were analyzed within a group of experiments conducted under the same agrotechnical conditions (A1 or A2) in Genstat version 21 (VSN International). Yield observations were analyzed using a linear model incorporating random effects of genotype, genotype × experiment interaction, and blocks within experiments. This model was used to calculate the yield score (BLUP) of the genotypes in each experiment, the average yield in the series, and heritability (Cullis et al. 2006). Also, for each set of experiments, an analysis was done using an additive main effect and multiplicative interaction (AMMI) model (Gauch 1992) to determine the coefficients of genotype stability (Purchase et al. 2000) which are the weighted distances of the genotypes from zero in the 2-dimensional plot of AMMI genotype scores.

An array of 8233 SNP markers was cut down to 7422 markers with known locations so that the structure of the population could be analyzed. These markers were further grouped into 705 linkage blocks based on a shared location within a 5-Mbp window (Tyrka et al. 2021a). In the same way, 8914 out of 11,117 silicoDArT markers were mapped on 21 CS wheat chromosomes, and 817 markers representing independent blocks of coupled markers were chosen. Markers with the lowest number of missing data in the blocks were used for the population structure analysis utilizing the STRUCTURE version 2.3.4 software (Pritchard et al. 2000). The admixture model was selected with 10,000 cycles and 1000 repetitions per cycle. The test was carried out over ten repetitions for ten possible subpopulations (*K* = 1–10). The *K* parameter was selected according to Evanno et al. (2005). The general (GLM) and mixed (MLM) linear models with PCA-based structure correction were used to determine the marker-trait associations using the TASSEL 5.0 (Ithaca, New York, NY, USA) (Bradbury et al. 2007). Benjamini-Hochberg (BH) method (Benjamini and Hochberg 1995) was used to adjust the *P*-values for allelic substitution effects for multiple tests. Associations were considered significant if the BH-corrected *P*-value was below 0.05, which usually meant that the original *P*-value was below 0.001. The Bonferroni corrected *P*-values for associations with silicoDArTs and SNPs were 0.0007 and 0.0006, respectively.

## Results

Significant variations and effects of the environment were found for all the traits studied (Table 2). The experimental design does not allow a direct comparison of the effects of applied nitrogen fertilization at A1 and A2 agrotechnical levels. The mean grain yields at A1 and A2 cultivation levels were 11.29 and 10.84 t·ha^−1^, respectively. At the A1 level, the average plant height was 102.2 cm, and the lodging score was 7.59. Retardant sprays were applied at the A2 level, resulting in a mean plant height of 97.6 cm and lodging of 7.07. Except for DTH, standard deviations from experiments at level A1 were lower than those at level A2 (Table 2). Experiments conducted on A1 showed higher heritability of GY compared to the A2 level (0.714 and 0.568, respectively); therefore, they may provide more stable data for GWAS and genomic prediction studies. Higher heritability values on the A1 cultivation level were also found for PH, LDG, and TKW (Table 2).

**Table 2:** Means, standard deviations, heritability (H), and F-values for phenotypic characteristics measured under A1 and A2 agronomic levels

Trait	Unit	Min	Max	Mean ± SD	F-values			*H*
					Genotype	Environment	GxE	
GY_A1	t·ha^−1^	5.14	14.40	11.29 ± 1.10	1.75^***^	57.02^***^	1.22^***^	0.714
PH_A1	cm	80.00	129.50	102.22 ± 7.61	13.02^***^	146.93^***^	2.38	0.908
DTH_A1	days	141.00	165.00	151.73 ± 4.43	3.62^***^	273.06^***^	2.46^*^	0.742
LDG_A1	Scale 1–9	1.00	9.00	7.59 ± 1.85	3.24^***^	49.42^***^	1.88^*^	0.784
TKW_A1	g	30.34	59.31	45.95 ± 4.44	5.09^***^	19.65^***^	0.97	0.806
GY_A2	t·ha^−1^	5.80	15.00	10.84 ± 1.31	1.63^***^	122.00^***^	2.12^***^	0.568
PH_A2	cm	70.00	130.00	97.56 ± 10.66	4.30^***^	1109.99^***^	1.45	0.876
DTH_A2	days	136.00	171.00	146.59 ± 3.12	5.43^***^	1005.45^***^	1.44^**^	0.777
LDG_A2	Scale 1–9	1.00	9.00	7.07 ± 1.98	3.44^***^	7.21^*^	0.42	0.649
TKW_A2	g	27.7	66.86	45.57 ± 8.04	2.43^***^	1518.75^***^	1.49	0.691

*SD* standard deviation, *GxE* genotype × environment interaction

^***^, ^**^, and,^*^ *p*-value below 0.0001, 0.001, and 0.01, respectively

**Fig. 2:**
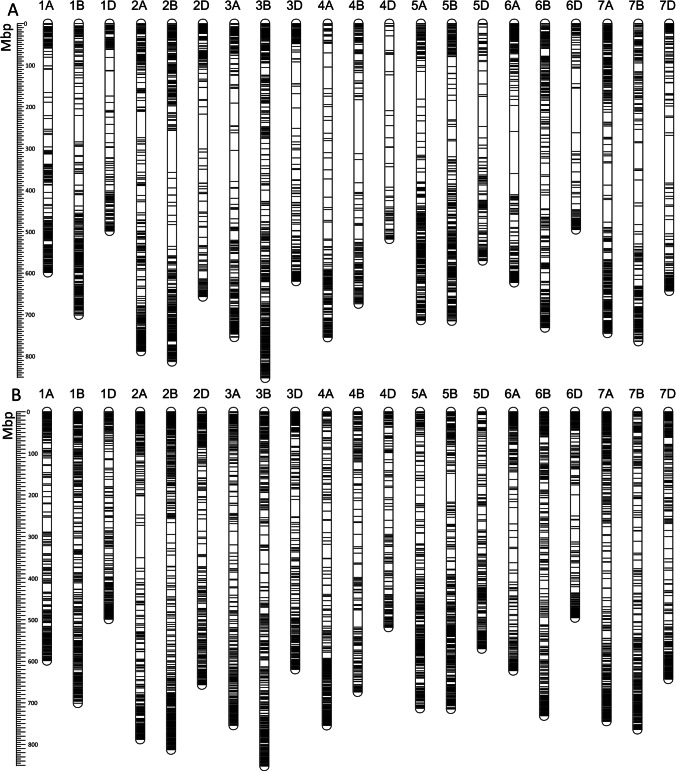
Physical distribution of selected 8233 SNP (**A**) and 11,117 silicoDArT markers (**B**) on wheat chromosomes (IWGSC RefSeq v 2.1). Seven chromosomes were numbered in A, B, and D genome. Mbp – millions of base pairs

DArTseq analysis yielded two types of markers, i.e., SNPs and PAVs (silicoDArT). Due to the different characteristics of these markers, they were used separately in the analysis. SNP markers were identified as polymorphisms in 69-bp long nucleotide sequences of DArTseq markers. SilicoDArT markers, on the other hand, refer to the presence or absence of an entire marker sequence in individual genotypes. PAVs may result from mutations of a genetic or epigenetic nature in the site recognized by the restriction enzymes used to generate the marker fragments. The distribution of both marker types in the wheat genome is not even, and differences in marker saturation on the chromosomes and genomes can be noticed (Fig. 2, Table S2).

The distribution of DArTseq markers on wheat chromosomes is not random, and a higher density can be observed in the distal regions. The silicoDArT markers cover the genome better than the SNP markers, which is best seen in the proximal regions of chromosomes 4B, 4D, 6A, and 6D (Fig. 2). At the sub-genome scale, most markers were mapped to chromosomes from the B, A, and then D genomes.

**Fig. 3:**
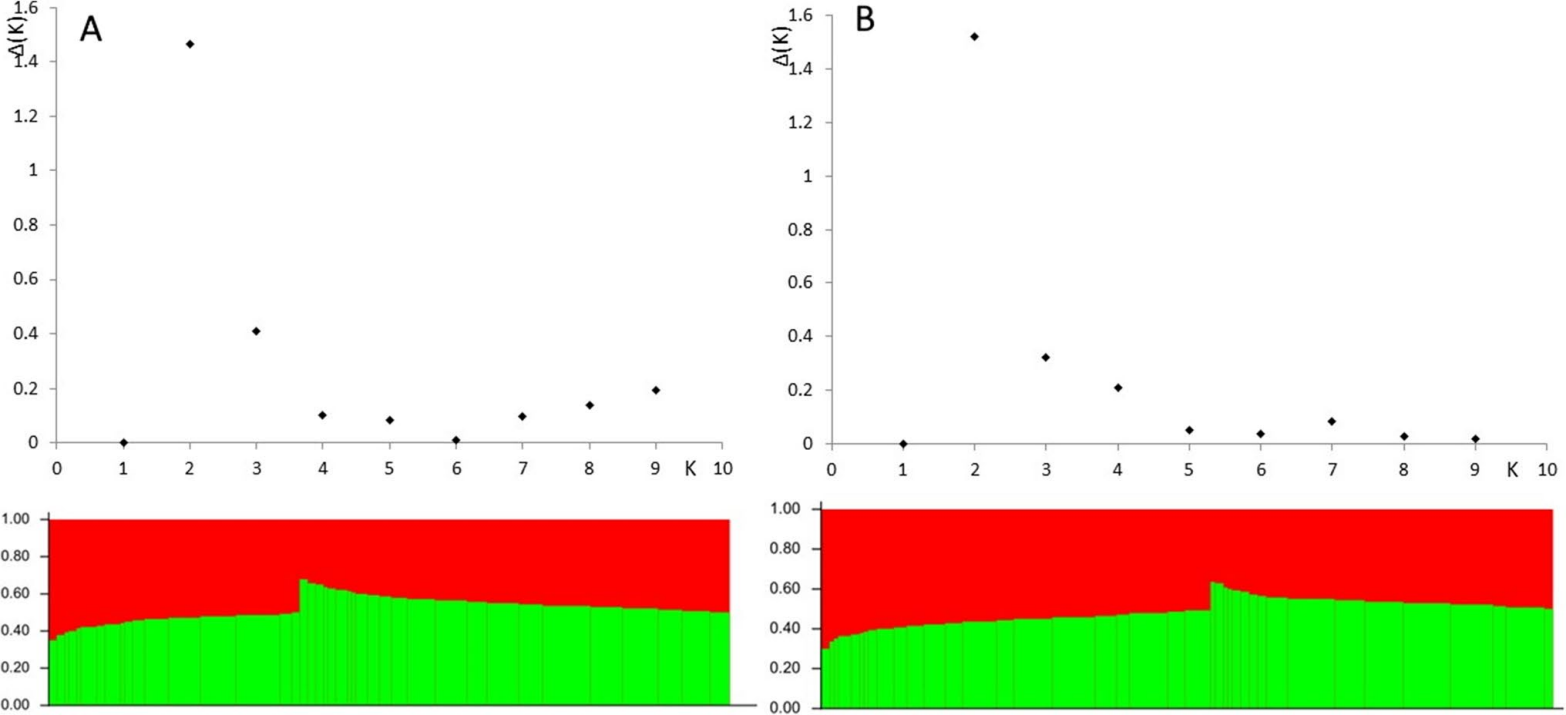
Number of populations identified with 1706 SNP (**A**) and 2383 silicoDArT (**B**) markers representing linkage blocks

To compensate for the uneven representation of particular regions of the genome, 1706 SNP and 2383 silicoDArT markers, spaced every 5 Mbp, were selected for the analysis of population structure (Fig. 3). It was found that the genotypes could be allocated to two subpopulations, while the detailed allocation of genotypes to these subpopulations based on SNP and silicoDArT markers overlapped for only half of the lines tested (Table S1).

**Fig. 4:**
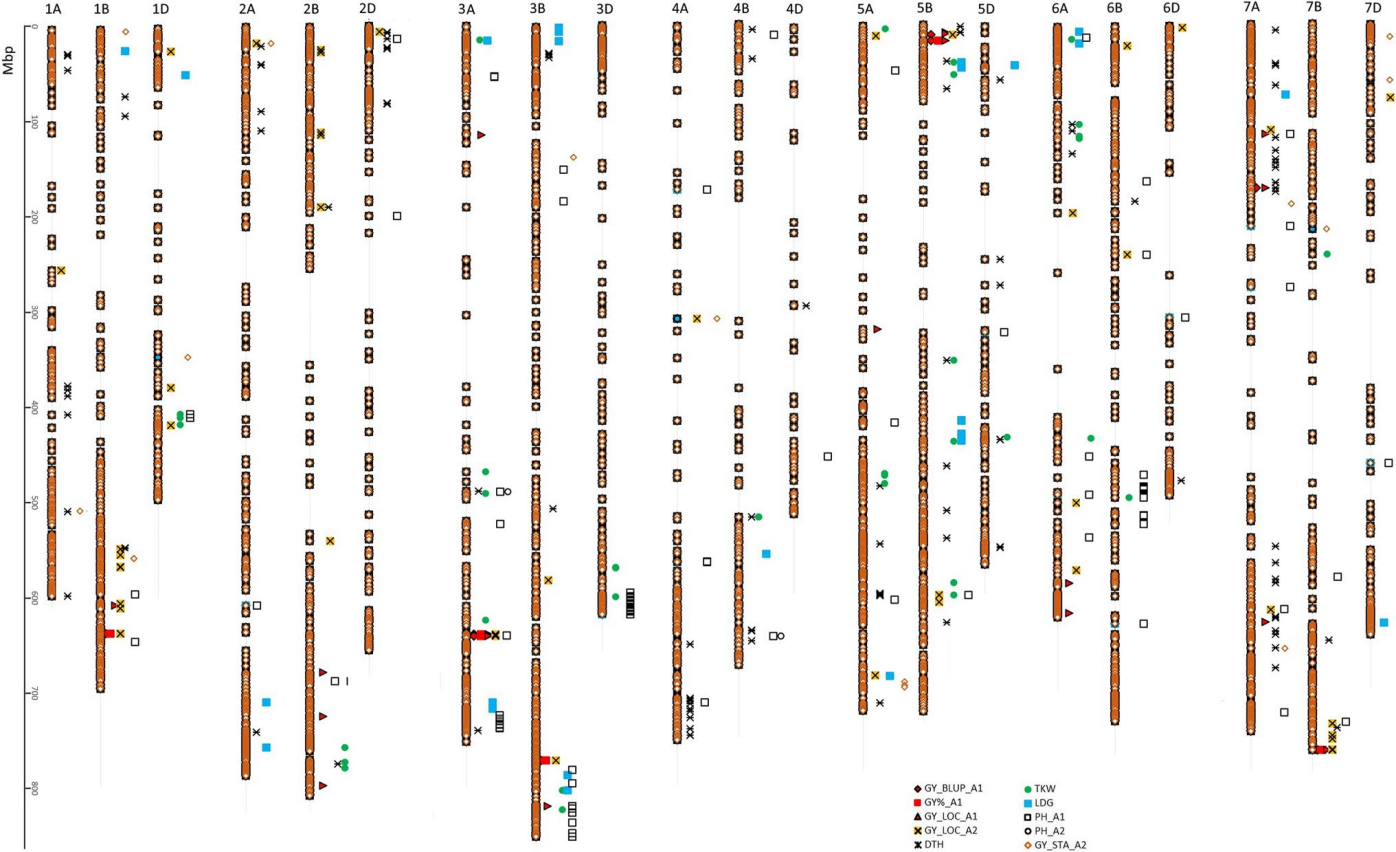
Distribution of MTA for grain yield (GY) for BLUP values, relative to standard (GY%), lines yield at selected locations at A1 and A2 level (GY_LOC_A1 and GY_LOC_A2, respectively), days to heading (DTH), thousand kernel weight (TKW), lodging (LDG), plant height (PH), and stability (GY_STA) on chromosomes covered with SNP markers

**Fig. 5:**
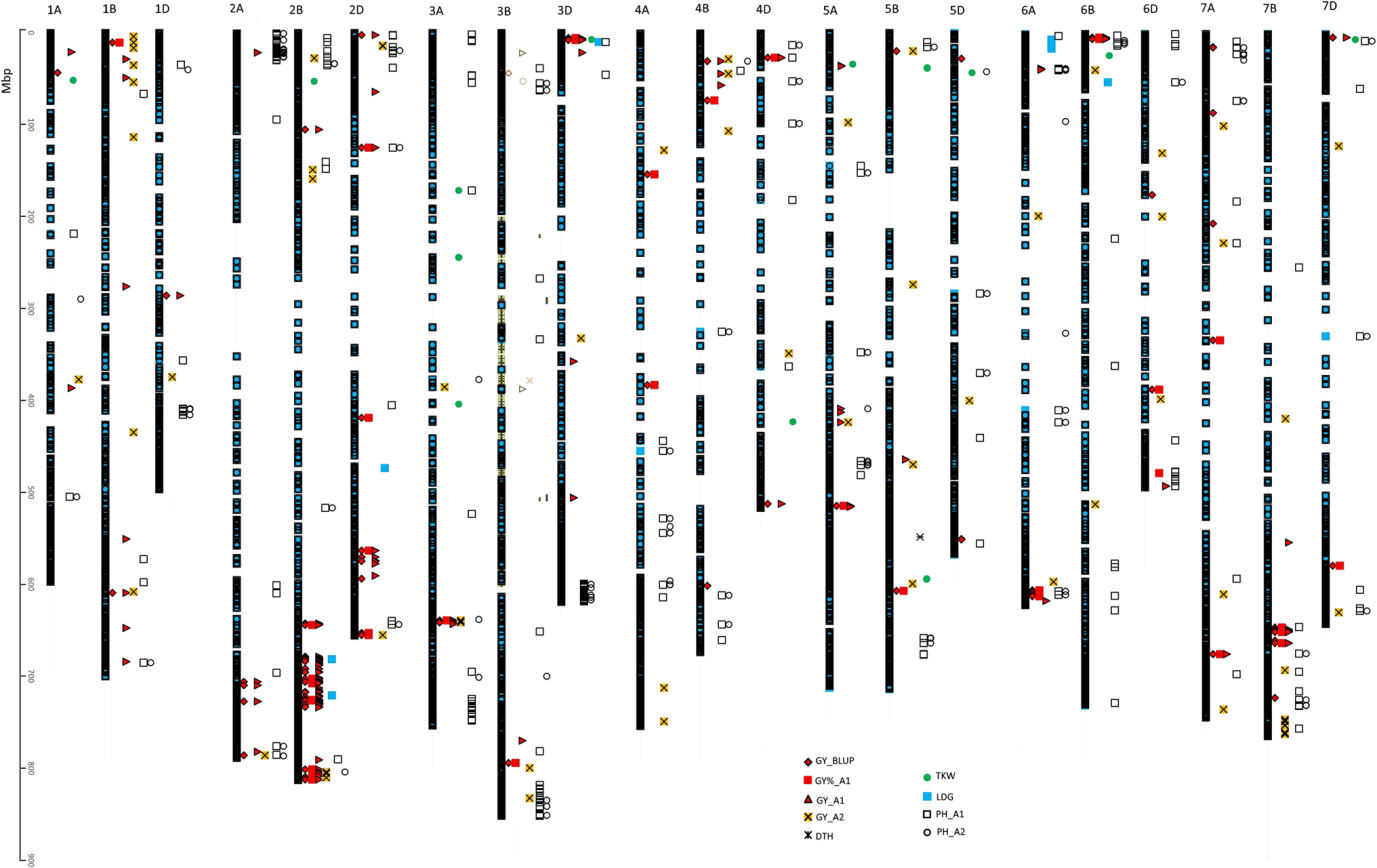
Distribution of MTA for grain yield (GY) for BLUP values, relative to standard (GY%), lines yield at selected locations at A1 and A2 level (GY_LOC_A1 and GY_LOC_A2, respectively), days to heading (DTH), thousand kernel weight (TKW), lodging (LDG), and plant height (PH) on chromosomes covered by with silicoDArT markers

Genotypic and phenotypic data were used to identify markers associated with grain yield and the other traits studied (Tables S3 and S4, Figs. 4 and 5). Yield analysis used BLUP values, yield relative to the standard (GY%), and stability results. No MTA was found for yield data at two locations (KOH and SMH) and respective BLUP values on the A2 fertilization level. In total, 95 and 422 MTAs with GY data were identified for SNPs and silicoDArTs, respectively (Table S5). MTAs for GY%, GY_BLUP, and site-specific yield that were mapped on common linkage blocks were used to choose the main loci responsible for GY variation in the selected panel of genotypes.

The universal loci significant for yield improvement were selected to better understand the main genetic factors influencing GY in an ongoing wheat breeding program. The main regions were selected when at least four independent MTAs for grain yield or stability coincided in a single linkage block. In total, 15 main regions were identified using the combined MTAs obtained for SNP and DArTseq markers (Table S5). The variation in GY_BLUP explained by the selected loci varied from 20.3% for *QGy.rut*-*3A* to 7.9% for *QGy.rut*-*6A*. Loci *QGy.rut*-*3D*, *QGy.rut-5B*, and *QGy.rut-6B* had pleiotropic effects. *QGy.rut-3D* shaped additionally other traits such as PH (9%), TKW (9.4%), and LDG (11.7%). *QGy.rut-5B* was simultaneously responsible for 15.9% of the variation in DTH, and *QGy.rut-6B* had a pleiotropic effect on PH (10% of variation). A single MTA (*QGy.rut-5A*) for stability was identified (Table 3).

**Table 3:** Main loci from linkage blocks responsible for variation of grain yield and stability. Number of significant MTAs in brackets

MTA	Flanking markers		Chromosome	Linkage block SNP/silicoDArT	Position (Mbp)		*R*^2^				Environments
					IWGSC_v1.0		GY_BLUP	GY_%STD	STA	Other traits	
*QGy.rut-1B*	1,235,724	4,410,248	1B	157/-	632.7	632.7	10.9 (3)	11.1 (1)	-		MOB
*QGy.rut-2B.1*	2,275,308	3,938,883	2B	-/548	634.4	636.1	8 (6)	8.2 (1)	-		NAD
*QGy.rut-2B.2*	3,020,780	3,030,699	2B	-/556–559	670.9	687.5	8.7 (28)	-	-		NAD, DED
*QGy.rut-2B.3*	1,100,592	1,097,050	2B	-/561–567	694.3	725.1	10.6 (51)	9.7 (5)	-		NAD, DED
*QGy.rut-2B.4*	3,532,864	1,090,370	2B	-/580–583	790.9	801.9	10.9 (13)	11 (4)	-		DED, KOH, POB, RAH, STH
*QGy.rut-2D.1*	1,118,056	1,002,393	2D	-/676–678	561.0	572.3	9.2 (6)	8.1 (1)	-		NAD, DED
*QGy.rut-2D.2*	1,129,191	1,105,580	2D	-/694	633.5	635.9	9 (5)	8.8 (2)	-		KBP
*QGy.rut-3A*	1,207,429	1,076,743	3A	563–564/797–798	639.7	641.9	20.3 (48)	19.2 (13)	-		NAD, KBP, MOB, POB, RAH, SMH, STH
*QGy.rut-3D*	1,087,696	1,266,639	3D	-/969–970	5.7	9.9	10.3 (12)	10.4 (8)	-	PH 9 (1), TKW 9.4 (1), LDG 11.7 (1)	DED, RAH
*QGy.rut-5A*	999,327	34,092,080	5A	1058/-	685.0	687.4	-	-	13.4 (4)		-
*QGy.rut-5B*	1,089,247	1,091,392	5B	1065–1067/1503	7.7	16.2	10.7 (6)	9.8 (1)	-	DTH 15.9 (4)	NAD, KBP, STH
*QGy.rut-6A*	3,951,541	3,533,315	6A	-/1809–1810	605.2	609.7	7.9 (6)	9.3 (5)	-	-	-
*QGy.rut-6B*	7,913,548	2,280,230	6B	-/1813–1814	4.9	9.1	9.1 (6)	9.7 (3)	-	PH 10 (3)	STH
*QGy.rut-7B.1*	1,272,443	3,020,355	7B	-/2257	641.9	644.4	11.1 (7)	11.6 (4)	-		RAH
*QGy.rut-7B.2*	1,254,272	3,022,330	7B	1640/2280	740.6	743.2	12.4 (8)	13.1 (1)	-		KBP, STH

*GY_BLUP* grain yield, *GY_%STD* grain yield relative to standards, *STA* yield stability, *DTH* days to heading, *PH* plant height, *TKW* thousand kernel weight, *LDG* lodging

MTAs for 183 SNP and 198 PAV loci were identified for heading time, lodging resistance, plant height, or thousand kernel weight (Table S6). These data were used to select 23 central regions corresponding with variation, mainly in PH, DTH, TKW, and LGD, with 18, 8, 6, and 2 linkage blocks, respectively (Table 4). Selected loci like *QPhen.rut-3D* and *QPhen.rut-2A* accumulated 76 and 44 MTAs and explained 15.8% and 10.3% of the variation in PH, respectively. In addition, two main loci (*QPhen.rut-5B.1* and *QPhen.rut-6A.1*) were found for variation in lodging.

**Table 4:** Main loci from linkage blocks responsible for variation in days to heading (DTH), plant height (PH), thousand kernel weight (TKW), and lodging (LDG). Number of significant MTA in brackets

MTA	Flanking markers		Chromosome	Linkage block SNP/silicoDArT	Position (Mbp)		*R*^2^ (%)			
					IWGSC_v1.0		DTH	PH	TKW	LDG
*QPhen.rut-1D*	1,117,337	2,309,352	1D	205/304	404.6	411.5	-	10.5 (9)	10.9 (2)	-
*QPhen.rut-2A*	4,404,733	1,272,923	2A	-/322–326	0.3	22.2	-	10.3 (44)	-	-
*QPhen.rut-2B*	1,109,361	4,410,315	2B	-/447	30.6	31.7	-	9.6 (5)	-	-
*QPhen.rut-2D.1*	2,244,477	1,121,891	2D	440/587	13.2	19.1	9 (1)	10.5 (7)	-	-
*QPhen.rut-2D.2*	7,352,485	4,991,692	2D	453/-	79.1	79.7	10.8 (3)	-	-	-
*QPhen.rut-3A*	3,954,418	1,123,465	3A	580–582/815–816	727.1	739.3	9.9 (1)	10.9 (11)	-	-
*QPhen.rut-3B.1*	992,877	4,405,430	3B	591/-	23.9	24.9	21.2 (4)	-	-	-
*QPhen.rut-3B.2*	1,673,925	1,167,112	3B	715/962–967	804.0	823.6	-	11.1 (21)	13.1 (1)	-
*QPhen.rut-3D*	997,166	997,104	3D	787–790/1072–1076	597.6	613.9	-	15.8 (75)	11.7 (1)	-
*QPhen.rut-4A*	1,159,667	1,092,932	4A	-/1159	601.3	601.3	-	8.6 (4)	-	-
*QPhen.rut-4B*	1,221,215	1,126,358	4B	900/-	519.4	519.5	9.5 (1)	-	11.6 (3)	-
*QPhen.rut-5A.1*	5,365,308	3,533,962	5A	-/1420	347.5	347.5	-	13.4 (3)	-	-
*QPhen.rut-5A.2*	1,137,905	985,861	5A	1018/1443	466.0	478.9	9.2 (1)	7.7 (7)	18.7 (2)	-
*QPhen.rut-5A.3*	1,219,158	1,128,511	5A	1040/-	589.6	592.0	19.6 (6)	-	-	-
*QPhen.rut-5B.1*	978,618	1,112,545	5B	1071/-	65.8	43.4	11.9 (1)	-	13.3 (4)	16.2 (5)
*QPhen.rut-5B.2*	1,112,093	1,100,263	5B	-/1608	658.5	658.6	-	8.4 (5)	-	-
*QPhen.rut-6A.1*	3,022,478	1,266,676	6A	-/1723–1724	1.7	7.0	-	5.3 (1)	-	13.3 (6)
*QPhen.rut-6A.2*	3,953,575	1,219,492	6A	-/1730	35.1	36.7	-	10.1 (9)	-	-
*QPhen.rut-6B*	5,323,859	1,054,930	6B	1363/-	476.1	477.7	-	8.9 (3)	-	-
*QPhen.rut-6D*	1,089,216	2,242,966	6D	-/2020	456.7	457.3	-	7.5 (3)	-	-
*QPhen.rut-7A.1*	1,127,215	1,094,572	7A	-/2028	20.7	22.1	-	5.6 (4)	-	-
*QPhen.rut-7A.2*	1,101,462	1,005,950	7A	-/2038	69.9	70.0	-	8 (5)	-	-
*QPhen.rut-7B*	1,120,511	4,992,001	7B	-/2273	720.6	720.8	-	8.9 (7)	-	-

*DTH* days to heading, *PH* plant height, *TKW* thousand kernel weight, *LDG* lodging

## Discussion

Genomic regions harboring selection signatures were different by over 80% between the European and Asian germplasm, suggesting independent improvement targets from the two geographic origins (Pont et al. 2019). Therefore, the selection of genotypes for association analyses depends on the research objective. Genetically diverse or segregating populations can be used to identify major loci determining complex quantitative traits. However, not all loci determining wide variation may be relevant for ongoing breeding programs. In the genetic background uniform for selected main genes, other genes are becoming more important. GWAS on elite lines from pre-registration experiments enables the identification of regions significant for yield improvement.

We identified a set of SNP and PAV markers for 15 main regions for yield improvement in ongoing winter wheat breeding programs. We used meta-analyses (Yang et al. 2021) to find four yield-related regions overlapping with meta-QTLs (Table 5). QTL 2B-5 was reported to affect the number of grains. Regions 3A-4 and 6A-8, with the genes *MOC2* and *OSGA20ox1*, determine variation in seed number, weight, and yield. Another QTL 7B-8 with the *Brd2* gene conditions seed number and weight (Yang et al. 2021). The location of *QGy.rut-5A* was consistent with the position of haplotype H20271, which is associated with variation in yield (Li et al. 2018), and *QYld.aww-5A* explained 2.3% of the variance (Garcia et al. 2019). SNP S2B_692461029 (*TraesCS2B01G495700*) affecting the number of grains was localized in the region corresponding to *QGy.rut-2B.4* (Pradhan et al. 2019).

**Table 5:** Main loci responsible for variation of grain yield and stability and corresponding genes, haplotypes, or QTLs

MTA	Chr	Region [Mbp]		Gene/haplotype/QTL	Reference
*QGy.rut-1B*	1B:	632.7	632.7		
*QGy.rut-2B.1*	2B	634.4	636.1		
*QGy.rut-2B.2*	2B	670.9	687.5	*TraesCS2B03G1238800*, *StSase*	This study
*QGy.rut-2B.3*	2B	694.3	725.1	*TraesCS2B01G495700*	Pradhan et al. (2019)
*QGy.rut-2B.4*	2B	790.9	801.9	2B-5	Yang et al. (2021)
*QGy.rut-2D.1*	2D	561.0	572.3	*TraesCS2D03G1048800*, *StSase*	This study
*QGy.rut-2D.2*	2D	633.5	635.9	Excalibur_rep_c102984_157	Dreccer et al. (2022)
*QGy.rut-3A*	3A	639.7	641.9	*TraesCS3A02G377600*, *MOC2*, 3A-4	Yang et al. (2021)
*QGy.rut-3D*	3D	5.7	9.9	*TraesCS3D03G0024300*, *SuSase*	This study
*QGy.rut-5A*	5A	685.0	687.4	H20271, *QYld.aww-5A*, *Rht12 TraesCS5A01G543100*, *GA2-β-dioxygenase*, *TaGA2ox-A14*	Li et al. (2018); Garcia et al. (2019); Sun et al. (2019); Ellis et al. (2005)
*QGy.rut-5B*	5B	7.7	16.2		
*QGy.rut-6A*	6A	605.2	609.7	*TraesCS4A02G319100*, *OsGA20ox16*, A-8	Yang et al. (2021)
*QGy.rut-6B*	6B	4.9	9.1	Ex_c3405_203	Dreccer et al. (2022)
*QGy.rut-7B.1*	7B	641.9	644.4		
*QGy.rut-7B.2*	7B	740.6	743.2	*TraesCS7B02G484200*, *Brd2*, 7B-8	Yang et al. (2021)

Wheat yield strongly depends on the efficient accumulation of starch in grains. Starch contributes to 60–75% of the total dry weight of the wheat grain (Sawaya et al. 1984). Starch biosynthesis involves enzymes necessary to produce sucrose in the photosynthesis process. Then, sucrose is transported to amyloplasts and metabolized to hexose phosphate. Hexose phosphate is a substrate for the biosynthesis of oil, protein, and starch. During endosperm development, most of the phosphate is used to produce starch. In amyloplasts, hexose phosphate is metabolized to ADP-glucose (Shewry 2009; Thitisaksakul et al. 2012). The activities of four key enzymes involved in sucrose-to-starch conversion, sucrose synthase (*SuSase*), adenosine diphosphate-glucose pyrophosphorylase (*AGPase*), starch synthase (*StSase*), and starch branching enzyme (*SBE*), were significantly correlated with the grain-filling rate (Zhang et al. 2011). The wheat sucrose synthase 2 gene (*TaSus2-2B*) affecting grain weight has also been identified (Jiang et al. 2011) on chromosome 2B at 179 Mbp. We found probable sucrose-phosphate synthase 4 (LOC123076775; 3D: 4,469,233.0.4477157) is close to *QGy.rut-3D*. Two loci coding starch synthase 3 (LOC100136992 2B: 698,067,030.0.698075303; LOC123054641 2D: 577,064,215.0.577073489) are localized in the regions of *QGy.rut-2B.2* and *QGy.rut-2D.1*, respectively.

Comparative analysis of yield-related traits revealed 145 meta-QTLs and candidate genes (Yang et al. 2021). About 40 genes associated with GY and related traits have been cloned (Liu et al. 2012; Rasheed et al. 2016; Nadolska-Orczyk et al. 2017), and functional markers have been converted to competitive allele-specific PCR (KASP) (Rasheed et al. 2016). However, some of these genes have already been established in modern lines. For example, no genetic differentiation was detected around the photoperiod regulation genes *Ppd-B1*, *Vrn-2*, and *Vrn-3* (Cavanagh et al. 2013). Most accessions carrying the favorable haplotype at these QTLs came from CIMMYT, with 95% of them also carrying the dwarfing allele at *Rht-B1* (Garcia et al. 2019). Other genes, *TaNMR-1B* and *TaCOL5-7B*, associated with yield increase in biparental populations have been cloned (Kan et al. 2020; Zhang et al. 2022) but not introduced to Polish breeding programs, and no significant MTAs were found in the respective regions.

Nine QTLs colocalized with regions identified in meta-analysis (Table 6) by Yang et al. (2021), including six (2A-2, 2B-2, 2D-2, 4A-2, 5A-3, and 6A-1) associated with kernel number, width, and yield. In addition, region *QPhen.rut-3A* corresponded to the IWA94 marker (3A 727.9–741.1) of a pleiotropic locus significantly associated with GY and six other yield-related traits (Li et al. 2019).

**Table 6:** Main loci responsible for variation of days to heading, plant height, thousand kernel weight and lodging, and corresponding genes, haplotypes, or QTLs

MTA	Chr	Region [Mbp]		Gene/haplotype/QTL	Reference
*QPhen.rut-1D*	1D	404.6	411.5		
*QPhen.rut-2A*	2A	0.3	22.2	*TraesCS2A02G000200*, *OsETR2*, 2A-2 *TraesCS2A02G027500*	Yang et al. (2021) Zanke et al. (2014)
*QPhen.rut-2B*	2B	30.6	31.7	*TraesCS2B02G048700*, *OsARG*, 2B-2	Yang et al. (2021)
*QPhen.rut-2D.1*	2D	13.2	19.1	*TraesCS2D02G034900*, *OsARG*, 2D-2 *TraesCS2D02G051800*, *Rht8*	Yang et al. (2021) Korzun et al. (1998)
*QPhen.rut-2D.2*	2D	79.1	79.7	*QMat3.aww-2A.2*	Garcia et al. (2019)
*QPhen.rut-3A*	3A	727.1	739.3	AX_111492146	Li et al. (2018)
*QPhen.rut-3B.1*	3B	23.9	24.9	3B-3, AX_109881378	Yang et al. (2021) Li et al. (2018)
*QPhen.rut-3B.2*	3B	804.0	823.6	*QTKW.td.ipbb*_3B.3, *TaCM*	Golabadi et al. (2011), Mangini et al. (2018)
*QPhen.rut-3D*	3D	597.6	613.9	3D-4, *TraesCS3D02G535700*, *MOC2*	Yang et al. (2021)
*QPhen.rut-4A*	4A	601.3	601.3	4A-2, *TraesCS4A02G319100*, *OsGA20ox1*	Yang et al. (2021)
*QPhen.rut-4B*	4B	519.4	519.5	Ra_c27465_569	Sukumaran et al. (2018)
*QPhen.rut-5A.1*	5A	347.5	347.5		
*QPhen.rut-5A.2*	5A	466.0	478.9	*QHD.td.ipbb*_5A.1	Peng et al. (2003)
*QPhen.rut-5A.3*	5A	589.6	592.0	5A-3, *TraesCS5A02G391800*, *OsMADS34*, AX_110518148, *Vrn-A1*	Yang et al. (2021) Li et al. (2018)
*QPhen.rut-5B.1*	5B	65.8	43.4		
*QPhen.rut-5B.2*	5B	658.5	658.6	*TraesCS5B02G486900*	Mokrzycka et al. (2022)
*QPhen.rut-6A.1*	6A	1.7	7.0	6A-1, *TraesCS6A02G017500*, *OsNR2*	Yang et al. (2021)
*QPhen.rut-6A.2*	6A	35.1	36.7		
*QPhen.rut-6B*	6B	476.1	477.7		Liu et al. (2018)
*QPhen.rut-6D*	6D	456.7	457.3	6D-4, *TraesCS6D02G377900*	Yang et al. (2021) Zanke et al. (2014)
*QPhen.rut-7A.1*	7A	20.7	22.1	7A-1, *TraesCS7A02G006600*, *OsFBK12*,* Rht7* S7A_18997640, *TraesCS7A01G040900*	Peng et al. (2011) Pradhan et al. (2019)
*QPhen.rut-7A.2*	7A	69.9	70.0		
*QPhen.rut-7B*	7B	720.6	720.8		Li et al. (2018)

Some loci with known genes such as *Rht-B1*, *Rht-D1*, *Ppd-D1*, *Ppd-B1*, *Ppd-A1*, *Vrn-A1*, *Vrn-D1*, and *Vrn-B1* have been routinely employed in marker-assisted selection (Garcia et al. 2019). For this set of genes, only the *QPhen.rut-5A.3* locus is located at the position of the *Vrn-A1* gene (NC_057806.1, 5A:589,259,335.0.589271309), while no significant effects were found for the remaining genes, which may indicate the fixation of these alleles in modern breeding lines. For example, we found no significant effect of *Rht24* localized on the 6A chromosome at position 413.7 Mbp (Würschum et al. 2017).

Markers connected with plant height may also be significant for grain yield. For example, *TaRht12* increases the grain number per spike and the effective tiller number and decreases thousand-grain weight (Chen et al. 2013). This gene significantly improved the elite winter wheat lines investigated (*QGy.rut-5A*, Table 5). Furthermore, markers Ex_c3405_203 (6B: 0.9 Mbp) and Excalibur_rep_c102984_157 (2D: 641.1 Mbp) associated with the lodging score corresponded to *QGy.rut-6B* (6B: 4.9–9.1 Mbp) and *QGy.rut-2D.2* (2D: 633–635 Mbp), respectively (Dreccer et al. 2022).

Achieving optimal plant height is of prime importance for the cultivars’ stability, productivity, and yield potential (Griffiths et al. 2012). Improvement in wheat yield during the Green Revolution was achieved through the introduction of reduced-height (Rht) dwarfing genes. More than 50 loci and 25 height-reducing genes have been detected for wheat (Yang et al. 2021; Muhammad et al. 2021; Mokrzycka et al. 2022). Lodging may contribute to a reduction in grain yields of up to 50% (Stapper and Fischer 1990) and a loss of bread-making quality (Berry et al. 2004). The unpredictable occurrence of lodging has made it difficult for breeders to select for lodging tolerance. Ultimately, diagnostic genetic markers would help improve standability in a breeding program (Dreccer et al. 2022). By adding the semi-dwarfing genes Rht-B1b and Rht-D1b to modern wheat cultivars (Wilhelm et al. 2013; Berry and Berry 2015), the risk of lodging has been cut down. We found no effects from loci in the region of these genes. However, the *TaCM* (triacetin 3′,4′,5′-O-trimethyltransferase-like) gene responsible for lodging tolerance (Ma 2009) was mapped to chromosome 3B in a position consistent with *QPhen.rut-3B.2*.

The loci and significant SNP markers from this study can be used to create high-yield varieties by pyramiding the advantageous alleles. The introduction of a few major genes/QTL as fixed effects in GS models increases the accuracy of genomic selection for quantitative traits (Bernardo 2014) if each gene contributes to ≥ 10% of the variance (Sehgal et al. 2020). However, such significant effects of QTLs are rarely identified for complex traits such as GY in a typical GWAS study (Sehgal et al. 2016; 2017). The significant MTAs found in this study show a change in the genetic variation of the tested elite germplasm. To improve yield gains, an optimized set of markers should be used.

## Supplementary Information

Below is the link to the electronic supplementary material.

Supplementary file1 (DOCX 669 KB)

Supplementary file2 (XLSX 11590 KB)

## Data Availability

All data underlying the findings described in the manuscript are fully available without restriction from corresponding author.
