# Correction to: Genome‑wide association mapping in elite winter wheat breeding for yield improvement

**DOI:** 10.1007/s13353-023-00760-0

**Published:** 2023-05-13

**Authors:** Mirosław Tyrka, Paweł Krajewski, Piotr Tomasz Bednarek, Kinga Rączka, Tadeusz Drzazga, Przemysław Matysik, Róża Martofel, Urszula Woźna-Pawlak, Dorota Jasińska, Małgorzata Niewińska, Bogusława Ługowska, Dominika Ratajczak, Teresa Sikora, Edward Witkowski, Ada Dorczyk, Dorota Tyrka

**Affiliations:** 1 Department of Biotechnology and Bioinformatics, Rzeszow University of Technology, Powstańców Warszawy 6, 35-959 Rzeszów, Poland; 2 Institute of Plant Genetics, Polish Academy of Sciences, Strzeszyńska 34, 60-479 Poznań, Poland; 3 Plant Breeding and Acclimatization Institute – National Research Institute, Radzików, 05-870 Błonie, Poland; 4 Małopolska Plant Breeding Ltd, Sportowa 21, 55-040 Kobierzyce, Poland; 5 Plant Breeding Strzelce Group IHAR Ltd, Główna 20, 99-307 Strzelce, Poland; 6 Poznań Plant Breeding Ltd, Kasztanowa 5, 63-004 Tulce, Poland; 7 DANKO Plant Breeders Ltd, Ks. Strzybnego 23, 47-411 Rudnik, Poland; 8 Plant Breeding Smolice Ltd, Smolice 146, 63-740 Kobylin, Poland

**Correction to: Journal of Applied Genetics (2023)**

10.1007/s13353-023-00758-8

The original article published with inverted author names. Family name was captured first instead of given name. This is now correctly presented here.

The original article has been corrected.

