# Supplementary figures and images for: Genome-wide association mapping in elite winter wheat breeding for yield improvement
Source: J Appl Genet. 2023 Apr 29;64(3):377–91. doi: 10.1007/s13353-023-00758-8 (PMC10457411; doi:10.1007/s13353-023-00758-8)

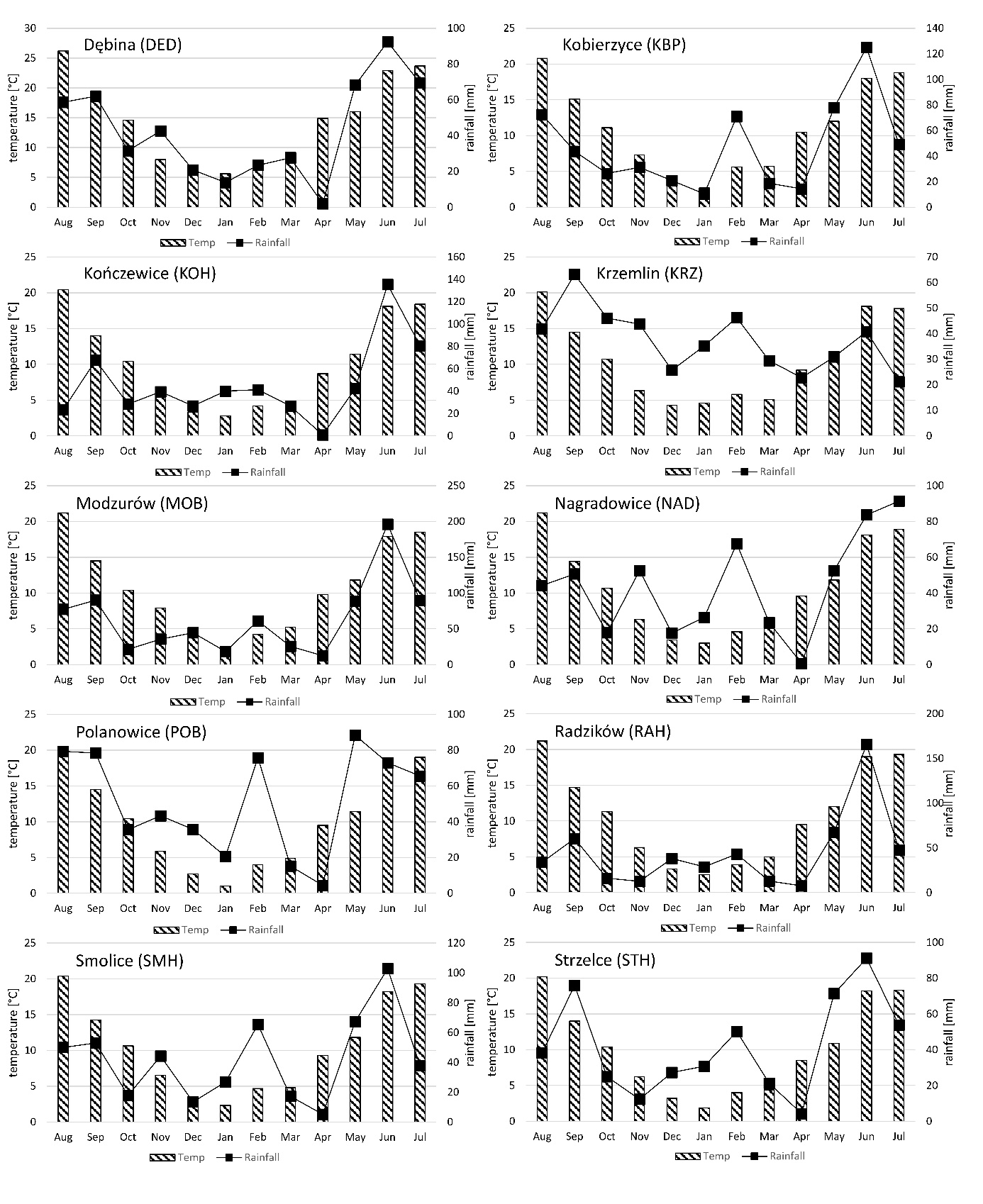


Figure S1. Mean temperatures and rainfalls since August 2019 to Jul 2020 in ten experimental stations.

Supplement: Supplementary file 1 — Supplementary file1 (DOCX 669 KB) [file 13353_2023_758_MOESM1_ESM.docx]
